# Effects of Psychological Factors on Modal Shift from Car to Dockless Bike Sharing: A Case Study of Nanjing, China

**DOI:** 10.3390/ijerph16183420

**Published:** 2019-09-14

**Authors:** Xinwei Ma, Ruiming Cao, Jianbiao Wang

**Affiliations:** 1 School of Transportation, Southeast University, Dongnandaxue Road 2, Nanjing 211189, China; 213160959@seu.edu.cn; 2 Architects & Engineers Co., LTD. of Southeast University, Sipailou 2, Nanjing 210096, China; 101010272@seu.edu.cn

**Keywords:** dockless bike sharing, modal shift, modified TAM, psychology factors, car

## Abstract

The emergence of dockless bike sharing in recent years has reduced the usage of private cars, especially usage for short-distance trips (within 2 km). In this paper, a modified technology acceptance model (TAM) is proposed to investigate from the psychological perspective drivers’ willingness to shift to dockless bike sharing. The modified TAM includes the perceived usefulness of dockless bike sharing, perceived ease-of-use of dockless bike sharing, perceived health of dockless bike sharing, attitudes toward dockless bike sharing, and willingness to shift to dockless bike sharing. Data are obtained through offline communications with car drivers. The results show that two-thirds of car drivers are willing to use dockless bike sharing in short-distance trips. Perceived health, perceived ease-of-use, and perceived usefulness have significant positive effects on people’s attitudes toward dockless bike sharing. As expected, people’s attitudes toward dockless bike sharing are positively correlated with their willingness to shift. Policy implications are discussed to prompt the modal shift from private cars to dockless bike sharing according to the results.

## 1. Introduction

The increasing number of private vehicles across the globe has caused great social and environmental problems such as noise, traffic congestion, and air pollution [1]. In response to this, bike-sharing systems have been adopted to reduce the use of private cars in short-distance trips [2,3,4,5]. Previous studies have shown that bike sharing is flexible, economical, and good for health; it helps cut down emissions, ease congestion, and reduce fuel usage; and it also supports multimodal transport connections [6,7,8,9].

Currently, bike-sharing systems operated worldwide can be divided into two categories: docked bike sharing and dockless bike sharing [10]. Docked bike sharing system requires users to rent bikes from and return them to designated docking stations. In contrast, dockless bike sharing system users can park bikes in physical or geo-fencing designated parking areas, or just scatter them in any areas accessible to other users [11].

There are already more than 2800 operational schemes in over 50 countries [12]. In China, bike-sharing systems first emerged in Beijing in 2005 [13]. However, unlike an information technology (IT)-based bike-sharing system, these schemes required users to return bikes to where they rented them. Thus, they failed soon. Later, the IT-based bike sharing service emerged in Hangzhou in 2008 [14], followed by Shanghai and Wuhan in 2009, and then Beijing in 2011 [15]. By December 2016, docked bike-sharing services were available in more than 400 Chinese cities. There were 890,000 bikes in 32,000 stations, and the number of users increased to 20,000,000 [16]. By the end of 2017, China had approximately 23 million dockless shared bikes and over 106 million users. The number of orders per day averaged 50 million [17]. The scale of both docked and dockless bike-sharing systems in China is larger than that of any other country [10,18,19].

Researchers have pointed out that both types of bike-sharing systems boost sustainable transportation all over the world. Particularly, these eco-friendly systems have affected modal shift on car use, public transit, and active transportation modes such as walking and bicycling [20]. Previous studies have focused on the effects of docked bike sharing on modal shift from active transportation modes [9,21,22], public transport [20,23], and car use [9,24,25]. There is a lack of evidence on how dockless bike sharing affects the modal shift on car use. More usage of dockless bike sharing instead of cars will benefit our living conditions. Understanding the modal shift in response to dockless bike sharing will help us design better policies and strategies to improve the bike-sharing service. This paper aims to figure out how psychological factors affect people’s willingness to shift from cars to dockless bike sharing. The following shows the focus of this paper:

(1) What are people’s responses regarding the willingness to shift from cars to dockless bike sharing in short distance (within 2 km), medium distance (2–5 km), and long-distance (more than 5 km)?

(2) How do psychological factors affect car drivers’ modal shift to dockless bike sharing?

The remainder of this paper is as follows. We review the relevant literature in Section 2 and introduce the city where the study is conducted in Section 3. In Section 4, we develop hypotheses and describes the data. The survey and model results are presented in Section 5. In Section 6 and Section 7, we present discussions and conclude this study, respectively.

## 2. Literature Review

Although the bicycle–car shift has been widely studied, the primary focus is on the modal shift from car to general bicycles or docked bike sharing. Due to the lack of studies on dockless bike sharing, this review section focuses on the comparative analysis of docked and dockless bike sharing, car modal shift in response to docked bike sharing, and the factors that affect the usage of dockless bike sharing.

### 2.1. Comparative Analysis of Docked and Dockless Bike Sharing

Several studies have analyzed travel pattern differences between docked bike sharing and dockless bike-sharing systems. Overall, docked bike-sharing users are more likely to be male, employed, rich, and higher educated, and have non-motorized vehicles [5,9]. Li et al. [26] and Xin et al. [27] discovered that dockless users were more likely to be male, higher educated, single, middle-income earners, and mobile network users. Chen et al. [28] revealed that older people were more willing to use docked bike sharing, while younger ones who were technology-sensitive prefer dockless bike sharing. The usage of both types of bike-sharing systems records a morning and afternoon peak period [29]. Chen et al. [28] concluded that for both modes, most users travel within 3 km in China. In addition, as the travel distance increases, the use frequency decreases. Gu et al. [30] discussed the recent development of bike sharing in China. It was suggested that cities with low cycling rates and high motor vehicles rates should adopt the docked bike sharing system. As a nonprofit program managed by the government, docked bike-sharing systems provide inexpensive yet valuable services [28]. The high quality and good riding experience of the docked sharing bikes satisfied their users [10]. Compared with the traditional docked system, the dockless bike-sharing system is integrated with smartphone applications, and is more flexible in terms of bike docking [30]. In terms of demerits, the docked bike-sharing system is often maligned, because users cannot find available bikes to rent or return bikes in a preferred destination [19]. In addition, the system requires users to rent and return bikes in fixed rental stations instead of offering door-to-door service [31]. For dockless bike sharing, bicycle maintenance is most problematic, because these dockless bikes are often unattended [28]. Moreover, these bikes are often oversupplied in areas with large populations, which hurts its economic sustainability, occupies urban space resources, harms the urban transport system, and causes visual pollution [17]. In addition, numerous dockless bike-sharing schemes fail because of huge financial or operational failures [24].

### 2.2. Car Modal Shift in Response to Bike Sharing

Bike sharing is not specifically designed to shift passengers from car use to active transportation [22]. However, it has reduced the usage of private cars and taxis [5], especially their usage in short trips in downtown areas [1,32,33]. Both Fan et al. [34] and Shaheen et al. [5] pointed out that the reductions in car use were partly because of the integration of bike sharing and public transit in the first and last mile of the trips. Interestingly, Yang et al. [35] concluded that the percentage of car ownership of metro bike-sharing users (48.8%) was twice as high as the percentage of car owners in the study area (19.7%). Previous studies showed that very few car trips were replaced by bike-sharing journeys. Fuller et al. [22] conducted two cross-sectional telephone surveys and proposed a calculation method to estimate the modal shift in response to bike-sharing systems. They observed that the percentage of modal shift from car to bike sharing was approximately 0.3–0.4%. Tang et al. [13] investigated the modal shift in response to bike-sharing programs in Chinese cities. They found that only 5.2%, 4%, and 0.46% of car uses were replaced by bike-sharing usage in Beijing, Shanghai, and Hangzhou, respectively. Statistical results showed that the percentages of modal shift from car to bike sharing were 3.6%, 2.0%, 2.1%, 1.9%, and 2% in Montreal, Toronto, Washington, D.C., Minneapolis-Saint Paul, and London, respectively [21,36]. However, the substitution rates of car by bike sharing in Minnesota, Melbourne, and Brisbane were relatively higher at 19%, 21%, and 19% respectively [21]. In the survey conducted by Yang [35], long-time driving, insufficient parking space, traffic jams, and high costs were regarded as the top reasons for the shift from private cars to bike sharing.

### 2.3. Factors Affecting Dockless Bike Sharing Usage

Several studies have analyzed the factors that affect dockless bike sharing. In socio-demographic aspects, previous studies found that dockless bike-sharing users are more likely to be male, younger, higher educated, single, and middle income earners [26,27,37]. Regarding occupation, most dockless bike-sharing users are company staff, followed by university students and the self-employed [27]. The usage peak of dockless bike sharing varies from weekdays to weekends, as well as in morning and evening rush hours, according to the research of Shen and Bao [38,39]. As for the effects of spatial distribution on dockless bike sharing, previous studies were inconsistent. Liu et al. [40] found that the usage of dockless bike sharing was higher in city central areas and business center surroundings. However, Shen et al. [38] found that the number of dockless bikes in central business districts was lower than that in populated peripheral residential areas. Zhou et al. [41] adopted a binary logistic model to explore the factors that affect the modal shift from walking and bus to dockless bike sharing riding. They found that the time of walking or bus was the most critical factor. Several studies also investigated the factors affecting the demand of dockless bike-sharing users, including weather conditions, the surrounding built environment, and bike infrastructure factors [38,42].

To summarize, previous literature has focused on the modal shift from car to docked bike sharing, but the impacts of dockless bike sharing schemes on car use are little discussed. As for the influencing factors, most researchers mainly analyzed the effects of socio-demographic and/or the built environments on dockless bike sharing usage. None of them examined the psychological determinants of the modal shift from car to dockless bike sharing. The current study advances the literature by developing a modified technology acceptance model (TAM) to examine the effects of psychological factors on car drivers’ willingness to shift to dockless bike sharing. The data used in the study came from a 2018 survey of 324 respondents in Nanjing, China.

## 3. Transportation Context in Nanjing: A Brief Overview

Located in the Yangtze River Delta economic zone, Nanjing is the political, economic, educational, and cultural center of Jiangsu Province, and has long been one of China’s most important cities [43]. With a total population of 8.2 million, Nanjing covers an area of 6587 km^2^ [44,45]. Multiple travel modes, including private cars, local buses, subways, taxis, private bikes, docked bike sharing, dockless bike sharing, and walking, are available in this city [46,47]. In 2016, the proportions of trips made by active mode were 55.3% (26.2% by walking and 29.1% by cycling respectively), 16.2% by bus, 10.8% by metro, and 15.5% by car (including car sharing and taxi) [48]. In 2016, the number of private cars reached 1.927 million, increasing year-on-year by 12.0% [48]. Nanjing Metro opened in 2005, and as of 2018, the metro system had 10 lines and 159 stations running on 393.628 km of track [49]. Available for transit systems, smartcards can be used for the metro, bus, docked bike sharing, ferry, and taxi in Nanjing. In 2016, the total number of bus passengers was 944.47 million, or a daily average number of 2.58 million, decreasing 7.7% compared to the previous year [48]. To ease traffic pressure and bring citizens great convenience, Nanjing launched the docked bike-sharing program in January, 2013. Classified as a third-generation bike sharing system, docked bike sharing adopts advanced technologies and management strategies such as smartcards for automated check-in and check-out [46]. After delivering a deposit of USD $35, users can ride docked shared bikes for free within two hours. Dockless bike sharing landed in Nanjing in January 2017. No docking stations are needed for this bike-sharing system. Bicycles can be parked in operational areas. With embedded Global Positioning System (GPS) tracking modules, riders can find and rent bicycles by using their smartphone. By the end of 2017, there were about 317,000 dockless shared bikes in Nanjing [48].

## 4. Methodology

In this part, TAM is introduced, and the hypotheses of this study are developed, followed by the survey design and data collection.

### 4.1. TAM and Hypotheses Development

TAM has been adopted in various technical contexts. Davis [50] proposed TAM in 1989 to explain and predict the usage of information technologies. According to TAM, user intentions are determined by two cognitive latent variables mediated through attitude: perceived usefulness and perceived ease-of-use. Perceived usefulness reflects the degree to which users feel that the information systems are useful, and perceived ease-of-use reflects whether users feel that the information systems are easy to use.

During the last several years, the performance of transportation has drastically enhanced partly due to new technologies. By using TAM, Rahman et al. [51] examined the acceptance of Advanced Driver Assistance Systems (ADAS), and Chen et al. [52] explored the effects of people’s green psychological cognition on docked bike sharing. Therefore, TAM is suitable for investigating—from the psychological perspective—car drivers’ willingness to shift to the new travel mode of dockless bike sharing. This study modifies the TAM with perceived health variables. This variable is proposed because cycling brings much greater physical benefits than driving.

The hypotheses are described below:

Previous studies showed that because driving involves little physical strength, it does no good to health [53]. In contrast, as a kind of active transportation mode, dockless bike sharing helps people do more exercise. Otero et al. [54] quantified the health risks and benefits of bike trips from European bike-sharing systems, and found that the health benefits from physical activities outweigh the health risks from traffic fatalities. Bike trips even help reduce the death rate. In addition, Šťastná et al. [55] concluded that cycling helps strengthen heart functions and reduce stress. Therefore, cycling benefits physical and mental health. In light of this, we propose that people who perceive the health value of dockless bike sharing have a positive attitude toward it:

**Hypothesis 1** **(H1).** Perceived health positively affects attitudes.

Bike-sharing systems make travel more convenient by reducing costs and freeing people from the responsibility associated with owning a bicycle [56]. Moreover, Li et al. [37] pointed out that dockless bike sharing is very helpful, because it offers door-to-door services and can be rented and parked at any time in operational areas. Such convenience makes people form positive attitudes to dockless bike sharing, and take it as a life-benefiting tool for travel:

**Hypothesis 2** **(H2).** Perceived ease-of-use positively affects attitudes.

**Hypothesis 3** **(H3).** Perceived ease-of-use positively affects perceived usefulness.

Bike sharing reduces greenhouse gas emissions, especially in city centers, relieves traffic jams, saves commuters’ time and money, and benefits the environment [57]. Dockless bike sharing is considered beneficial to travelers and the society as a whole. It will attract residents and prompt them to adopt it for its usefulness. Thus, the following hypotheses are developed:

**Hypothesis 4** **(H4).** Perceived usefulness positively affects attitudes.

**Hypothesis 5** **(H5).** Perceived usefulness positively affects people’s willingness to shift.

Attitudes toward dockless bike sharing in this study refer to the extent of a good or bad impression that the dockless bike sharing leaves on people. The relationship between attitudes and intention was explained in previous research [58,59]. We propose that people with positive attitudes to dockless bike sharing system are more likely to shift to it:

**Hypothesis 6** **(H6).** Attitudes positively affect people’s willingness to shift.

**Figure 1:**
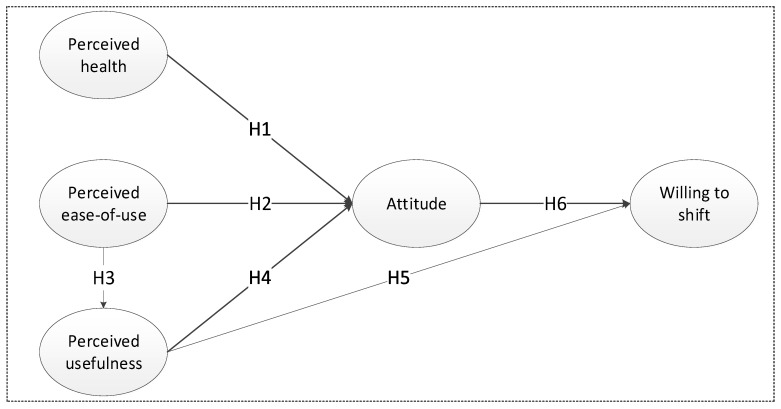
The theoretical framework of the modified technology acceptance model (TAM).

The theoretical framework of the modified TAM is shown in Figure 1.

### 4.2. Survey Design

The survey focuses on car drivers. The first part of the survey is about the socio-demographic information of participants, including age, annual income, gender, education, and occupation. The second part involves psychological questions such as people’s perceived usefulness, perceived ease-of-use, perceived health, attitudes, and willingness to shift from car to dockless bike sharing. The five-point Likert scale has been adopted in many behavioral intention studies [60,61]. In this survey, psychological questions are designed with a five-point Likert scale from 1 to 5, with 1 indicating a high degree of inconsistency and 5 indicating a high degree of consistency. Each psychologically relevant variable and their associated items are described as follows.

#### 4.2.1. Perceived Usefulness

Perceived usefulness (PU) refers to users’ belief that using dockless bike sharing can benefit their life. Three items are obtained from the research of Cheng and Huang [62] and Chen et al. [58]. They are PU1: Using dockless bike sharing will improve the living environment; PU2: Using dockless bike sharing will reduce travel time; and PU3: Using dockless bike sharing will reduce travel costs.

#### 4.2.2. Perceived Ease-of-Use

Perceived ease-of-use (PEOU) is the degree to which a person believes that using dockless bike sharing would be free of effort. The whole process can be simplified into these aspects: How easy or difficult it is to become a member of the dockless bike-sharing system, and to rent and return a dockless shared bike [50,52]. Adapted from Chen [58] and Davis et al. [50], three items are PEOU1: It is easy to become a member of dockless bike-sharing systems; PEOU2: It is easy to rent a dockless shared bike; and PEOU3: It is easy to return a dockless shared bike.

#### 4.2.3. Perceived Health 

Perceived health (PEH) is the psychological perception that individuals believe dockless bike sharing benefits their health. According to Šťastná et al. [55], the statements are PEH1: Dockless bike sharing promotes physical health; and PEH2: Dockless bike sharing helps relieve psychological stress.

#### 4.2.4. Attitudes

Attitudes (A) refer to the extent of a good or bad impression that dockless bike sharing leaves on people. According to Ajzen [63] and Borhan et al. [64], the statements are A1: The concept of dockless bike sharing is good; A2: The riding experience of dockless shared bikes is good; and A3: Illegal parking of dockless shared bikes brings lots of problems to the city. 

#### 4.2.5. Willingness to Shift

People’s willingness to shift (WTT) refers to their intention to shift from cars to dockless bike sharing. It varies in different travel distances. According to Fuller et al. [22], trips within 5 km are particularly amenable to active transportation. Cole et al. [65] pointed out that the travel distance of active transportation ranges from 1.6 km to 2 km. In light of this, this paper divides the travel distance into three categories: short distance (within 2 km), medium distance (2–5 km), and long-distance (more than 5 km). The statements are as follows. WTT1: For a short-distance trip, I am willing to shift from using a car to dockless bike sharing; WTT2: For a medium-distance trip, I am willing to shift from using a car to dockless bike sharing; and WTT3: For a long-distance trip, I am willing to shift from using a car to dockless bike sharing.

### 4.3. Data Collection

The questionnaire was delivered by 12 graduates in May and June, 2018. Respondents were well informed of the purpose of the study and were willing to cooperate. Data were collected from car drivers at parking lots and car-washing rooms in the main urban area of Nanjing during morning and evening peak hours. A total of 353 questionnaires were collected, of which 324 (91.7%) were valid. According to Kline [66], a minimum sample size of 200 is required to reduce bias to an acceptable level for any type of structural equation modeling estimation.

## 5. Results

The results are presented in three components. Firstly, the survey sample in terms of socio-demographics is described, and the willingness to shift in different travel distances is discussed. The second part reports the reliability and validity of the modified TAM, and then the hypothesis testing.

### 5.1. Descriptive Statistics

The descriptive statistics of sample composition are presented in Table 1. Car drivers were surveyed randomly, including 187 men (57.7%) and 137 women (42.3%). Out of all the participants, 6.2% had an education level below a bachelor’s degree, 261 participants (80.5%) had a bachelor’s degree, and 43 participants (13.2%) had an education level higher than a bachelor’s degree. According to the survey, 35.5% of the respondents are aged between 20–29, and 36.4% are aged between 40–49. Regarding income, 7.1% of participants earned less than 30,000 RMB ($US4485) a year, 9.9% were high-income earners with an annual income of over 200,000 RMB ($US29,900), and 83% of participants had an annual income from 30,000 RMB ($US4485) to 200,000 RMB ($US29,900). Regarding employment, 38.9% of respondents are civil servants, followed by private company staff (28.7%).

Respondents’ willingness to shift their mode of transport for different travel distances are classified into negative responses (extremely unwilling, relatively unwilling), neutral responses (general), and positive responses (relatively willing, extremely willing). Most of the respondents—207 (64.0%)—gave positive responses to shift for short-distance trips (within 2 km), 127 (39.2%) were willing to shift for middle-distance trips (2–5 km), and only 60 (18.6%) were willing to shift for long-distance trips (>5 km). Seventy respondents (21.6%), 117 (38.1%), and 84 (26.0%) gave neutral responses for short-distance trips, middle-distance trips, and long-distance trips, respectively. The proportion indicates that two-thirds of car drivers are willing to shift to dockless bike sharing for short-distance trips. As the travel distance increases, people’s willingness to shift to dockless bike sharing drops drastically. It is noted that nearly 40% of respondents gave neutral responses (defined as potential shift travelers) to shift for middle-distance trips, which is a ratio that is higher than those for short-and long-distance trips.

### 5.2. Reliability and Validity Assessment

The modified model shown in Figure 1 is evaluated using the visual tools provided by statistical software AMOS. The reliability and validity of the constructs are firstly tested. Table 2 shows that the factor loading of all the items is higher than 0.5 [58]. The value of composite reliability (CR) ranges from 0.751 to 0.816, and the Cronbach’s α value of the constructs ranges from 0.692 to 0.812. All of these values are around 0.70 [67], indicating that the model reliability is acceptable.

Average variance extracted (AVE) is used to test the construct validity. Ranging from 0.505 to 0.640, all the AVE scores are above the benchmark value of 0.50 [58]. Thus, the convergent validity is achieved. In addition, the discriminant validity requires testing. The square roots of all dimensions’ AVEs in Table 2 (ranging from 0.710 to 0.800) are higher than all the correlation coefficients in Table 3 (ranging from 0.000 to 0.369), ensuring the discriminant validity of this model [58].

### 5.3. Hypothesis Testing

The modified TAM is evaluated by using AMOS. The chi square ratio is 2.030, which is below the desired cutoff value of 3.000. The standardized root mean residual (SRMR) is 0.070, which is below the desired cutoff value 0.100. The root mean squared error of approximation (RMSEA) is 0.056, which is lower than 0.080. The Normed Fit Index (NFI) is 0.923, and the Comparative Fit Index (CFI) is 0.943, both of which are greater than 0.900. To summarize, the structural model has a good fit.

**Figure 2:**
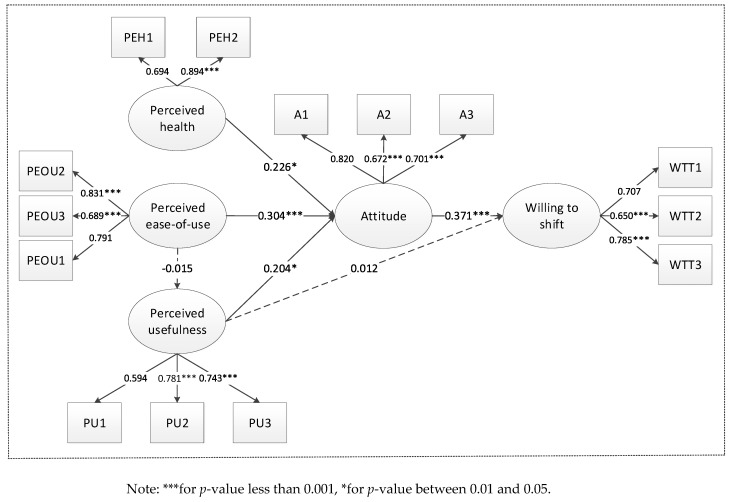
The results of the full model.

Figure 2 shows the path analysis for the model. As is shown in the diagram, four out of six hypotheses (H1, H2, H4, and H6) are confirmed. Specifically, the relationships between perceived health and attitudes (β=0.226,p<0.05), perceived ease of use and attitudes (β=0.304,p<0.001), as well as perceived usefulness and attitudes (β=0.204,p<0.01) are all significantly positive, hence supporting H1, H2, and H4. However, perceived ease-of-use has no statistically significant effect on perceived usefulness (β=−0.015,p>0.05). Thus, H3 cannot be supported. Perceived usefulness exerts almost insignificant influences on the willingness to shift (β=0.012,p>0.05), which means that H5 is not supported. As expected, the relationship between attitudes and willingness to shift is significant (β=0.371,p<0.001), thus confirming H6.

Perceived health has a positive effect on attitudes. This indicates that perceived health affects attitudes toward dockless bike sharing. This result is consistent with the finding of Otero et al. [54], who concluded that bike-sharing systems can promote people’s health and prevent illness. People nowadays are under unprecedented pressure from work and have little time to exercise. Although dockless bike sharing is mainly used for commuting, it is also popular for exercising. As people become more aware of their health, they hold positive attitudes toward dockless bike sharing.

Consistent with previous TAM studies [60], perceived ease-of-use has a strong positive influence on travelers’ attitudes. It seems that people are more likely to accept things that bring them greater convenience. The ease-of-use of dockless shared bikes makes its usage easier than other travel tools such as docked shared bikes or shared cars. Thus, people who prefer dockless bike sharing due to its ease-of-use characteristic are more likely to prefer this travel mode.

Perceived usefulness significantly affects the attitudes toward dockless shared bikes. As a useful alternative mode for travel, dockless bike sharing is eco-friendly, and it can relieve traffic congestion. It is also cheaper than other transport modes for users to complete their trips. This is why people hold positive attitudes toward it. However, the effect of perceived usefulness on willingness to shift is insignificant. This finding is inconsistent with the conclusion of other research that perceived usefulness directly affects behavioral willingness [50,52]. This may be because long travel distances make people less willing to shift to dockless bike sharing. Although dockless bike sharing is good for the living environment, travel distance is a more dominant factor in many travel situations [68]. In other words, dockless shared bikes are less efficient than cars and other motorized tools in long-distance travels, especially for commuting purpose.

There is no significant relationship between ease-of-use and usefulness. This contradicts with the result of Li et al. [37], who stated that flexible stations help improve the perceived usefulness of dockless bike sharing. However, in this study, the result is quite reasonable. For instance, people are less likely to use dockless shared bikes when they travel out of the service area or when they travel with children and the elderly.

Positive attitudes make people more willing to shift to shared bikes, which is in line with previous research on attitude–behavior relationships and travel behavior [58,59]. More and more people hold positive attitudes toward dockless bike sharing due to its advanced technology. Even if some have not used bike sharing, they could still know the benefits of the shared bikes. This obviously increases their likelihood to shift to dockless bike sharing.

## 6. Discussion

This research explored the psychological factors affecting drivers’ willingness to shift to dockless bike sharing. Here, we make policy suggestions for encouraging car drivers to use the dockless bike-sharing system. Based on our findings, several key points are discussed.

Firstly, more than 60% of car drivers gave positive responses to shift to dockless bike sharing for trips within 2 km. This finding echoes previous studies that bike sharing has become increasingly popular as an alternative active transportation mode to car use in short-distance trips [69,70]. Bike sharing is seen as a way to replace private cars in short-distance trips, because short car trips and bicycle trips in central downtown areas share similar characteristics. Park et al. [1] stated that exhaust emissions are serious in car trips less than three miles. Short-distance car trips require more fuel than long-distance trips, since they are more likely to be made in urban areas with cold engines. Therefore, the use of a dockless shared bike instead of a car for short-distance trips may significantly benefit the environment. Mackett et al. [71] reported that the convenience and time-saving merits make the car the dominant travel tool in short-distance trips. Jensen et al. [72] calculated a 13% reduction in travel time when using a shared bike compared to using a car for the same trip. In order to encourage users to use a dockless shared bike instead of a car for short-distance trips, dockless bike-sharing companies can design price strategies to adjust users’ travel behaviors. For instance, they can design more preferential plans to attract car users and make them realize that a dockless shared bike is more efficient, economical, and competitive than a car for short trips, especially during peak traffic periods and in urban areas [73]. In addition, dockless bike-sharing companies should improve their services. Residents have repeatedly complained about demand–supply imbalances of dockless bike-sharing services near residential communities, office areas, and metro stations during rush hours [30]. It is suggested that dockless bike-sharing companies should launch more shared bikes near those places during rush hours.

Previous research has found that bike-sharing services bring great health benefits [2,74]. In line with this finding, we propose that perceived health promotes the modal shift to dockless bike sharing. Park et al. [1] indicated that bike sharing helps citizens to live healthier and more pleasant lives by reducing their car use. Dockless shared bikes can replace cars in some trips, engaging people in physical exercises that are necessary for their daily life. Therefore, it is suggested that dockless bike-sharing companies design bikes more suitable for exercising and launch more dockless shared bikes near residential areas and workplaces to encourage commuters to use them for improving their physical and mental health. 

Both perceived ease of use and perceived usefulness have positive and statistically significant effects on attitudes, which is consistent with prior research by Chen et al. [75], Lee et al. [76], Yu et al. [77], and Cheng et al. [74]. This result implies that bike-sharing users will form positive attitudes toward these systems when they have experienced their benefits during usage. Particularly, the effect of perceived ease-of-use on attitudes is higher than that of perceived usefulness (0.304 versus 0.204) (see Figure 2). This is reasonable because the perceived ease-of-use is a fundamental requirement for the design of dockless bike-sharing services [74]. To improve the perceived ease-of-use, dockless bike-sharing companies should simplify the registration process and enable mobile payment such as Wechat Pay and Alipay to facilitate travelers to use shared bikes. Meanwhile, an effective mechanism for supervision, credit, and complaints feedback should be established to improve the dockless bike-sharing services. Designing diverse bicycles may help to reduce the age cohort and gender gap and attract more tourists. In order to make dockless bike sharing friendlier to elders, they should develop specialized smartphone applications. Operating companies are supposed to dispatch bikes in time to make it easier for users to find them. At the same time, relevant government departments should make legislative plans to regulate parking areas to make the dispatching process more efficient. In order to enhance the perceived usefulness of dockless bike-sharing services, the government should spread the benefits of the modal shift to dockless bike sharing, such as improving public transport connectivity, saving travel time, and reducing air pollution. In this way, it can strengthen public’s perception of the usefulness of dockless bike sharing [74].

Finally, positive attitudes make people more willing to shift to dockless bike sharing. Some strategies should be proposed to improve the impression of dockless bike sharing on people and attract more car users. Dockless bike sharing is popular due to its flexibility, convenience, and door-to-door services [37]. However, the rapid expansion of dockless bike sharing causes serious oversupply [78], which brings many troubles such as disorderly parking. It is suggested that dockless bike-sharing companies should launch bikes based on the predicted usage demand. On the other hand, they should apply used electronic fences and financial penalties to regulate users’ parking behaviors, thereby alleviating disorderly parking and gaining public goodwill. In addition, repairing damaged dockless shared bikes in time can leave a good impression on users. The maintenance of bicycles is one of the biggest problems, because they are often unattended [28]. Bike malfunctions will reduce user satisfaction and lower their loyalty [16]. Therefore, it is necessary to improve the quality of dockless shared bikes and strengthen their maintenance. Meanwhile, dockless bike-sharing companies should install lighting devices to improve the safety performance of their bicycles for riding at night. Previous studies have identified that a lack of bike-friendly infrastructure is one of the main barriers for bike-sharing usage [2,3]. The government should improve the bike infrastructure, prohibit car parking in exclusive bicycle lanes, and support facilities such as bike lanes, parking lots, and bike signals to make citizens feel safe and the bicycles more attractive [10,79,80]. Finally, both the government and dockless bike-sharing companies should invest in spreading the benefits of the bikes. In this way, people can develop good attitudes toward bike-sharing systems [10,74].

## 7. Conclusions

As a carbon-free travel mode, bike sharing can significantly reduce air pollution [81]. It can also improve public health by engaging people in physical exercise. A modal shift from car use to bike sharing brings immediate health benefits [82]. Besides the benefits to air quality and health, bike sharing also helps to reduce traffic noise, alleviate congestion, improve the urban environment, and contribute to a low carbon economy [83,84].

This study aims to explore the effects of psychological perceptions on people’s willingness to shift from cars to dockless shared bikes. A modified TAM involving psychological perceptions (perceived health, perceived usefulness, perceived ease-of-use, attitudes, and willingness to shift) is constructed based on the TAM. Data are obtained from a survey among 324 car drivers in Nanjing. The survey result shows that two-thirds of car drivers are willing to shift to dockless bike sharing for short-distance trips.

The results confirm four out of the six proposed hypotheses (H1, H2, H4, and H6). Perceived health, perceived ease-of-use, and perceived usefulness have significant positive effects. As expected, people’s attitudes toward dockless bike sharing is positively correlated with their willingness to shift. However, H3 and H5 are not supported, which means that perceived ease-of-use is poorly associated with perceived usefulness, and that perceived usefulness does not directly or significantly affect willingness to shift.

This study can provide important policy implications for government agencies and dockless bike-sharing companies to improve the bike-sharing services, especially in congested cities. By applying the dockless bike-sharing service, they can improve eco-friendly transportation systems and reduce the use of private cars.

This study has several limitations that need to be addressed in future research. Barbour et al. [69] pointed out personal attributes (such as gender and age groups) are key determinants to cycling. Thus, socio-demographic variables can be considered by getting a bigger sample size and building a multi-group structural equation model. In addition, Shen et al. [38] indicated that dockless bike sharing is also affected by weather conditions such as air temperature and precipitation. Future research should examine how weather factors affect the modal shift from private cars to dockless shared bikes. Moreover, a comparison study of docked and dockless bike sharing could be conducted to identify how these two travel modes affect car drivers’ willingness to shift. The study can also be improved by conducting a comparative analysis with survey data from smaller cities. Finally, electric dockless bike sharing is gaining popularity in many countries. With a higher speed and a higher degree of comfort than conventional bicycles and sharing bikes, electric dockless bike sharing could perhaps encourage some people, especially the elders, to use the service [2]. It is necessary to explore the factors that affect the modal shift from cars to electric dockless shared bikes.

## Figures and Tables

**Table 1:** Demographic characteristics.

Characteristics	Number	Ratio (%)	Characteristics	Number	Ratio (%)
**Gender**			**Education**		
Male	187	57.7	High school	20	6.2
Female	137	42.3	College/University	261	80.5
**Age (In Years)**			Graduate institute	43	13.3
<20	5	1.5	**Occupation**		
20–29	115	35.5	State-owned enterprise staff	43	13.3
30–39	79	24.4	Private company staff	93	28.7
40–49	118	36.4	Civil servant	126	38.9
>50	7	2.2	Self-employed	31	9.6
**Annual Income ($US)**			Retirement	6	1.9
<4485	23	7.1	Student	11	3.4
4485–7475	83	25.6	Other	14	4.2
7475–14,950	140	43.2			
14,950–29,900	46	14.2			
>29,900	32	9.9			

**Table 2:** Statistics of the measurement model. AVE: average variance extracted, CR: composite reliability.

Constructs	Items	Item Description	Factor Loading	Cronbach’s α	CR	AVE	Square Root of AVE
Perceived usefulness	PU1	improve living environment	0.594	0.719	0.751	0.505	0.710
	PU2	reduce travel time	0.781				
	PU3	reduce travel cost	0.743				
Perceived ease-of-use	PEOU1	ease of returning a dockless bike	0.791	0.812	0.816	0.597	0.773
	PEOU2	ease of renting a dockless bike	0.831				
	PEOU3	ease of being a member of a dockless bike-sharing system	0.689				
Perceived health	PEH1	improve physical health	0.694	0.765	0.778	0.640	0.800
	PEH2	relieve psychological stress	0.894				
attitudes	A1	attitudes to the concept of dockless bike sharing	0.820	0.717	0.776	0.538	0.733
	A2	attitudes to riding experience	0.672				
	A3	attitudes to illegal parking	0.701				
Willing to transfer	WTT1	shift willingness in short distance	0.707	0.692	0.758	0.513	0.716
	WTT2	shift willingness in middle distance	0.650				
	WTT3	shift willingness in long distance	0.785				

**Table 3:** Means, standard deviation, and correlations.

	M	SD	Perceived Usefulness	Perceived Ease-of-Use	Perceived Health	Attitudes	Willing to Transfer
Perceived usefulness	4.014	0.983	1				
Perceived ease-of-use	3.543	1.119	0.000	1			
Perceived health	3.817	1.026	0.000	0.193	1		
Attitude	3.675	1.011	0.213	0.297	0.292	1	
Willing to transfer	3.050	1.236	0.088	0.109	0.108	0.369	1
